# Genes polymorphism as risk factor of recurrent urolithiasis: a systematic review and meta-analysis

**DOI:** 10.1186/s12882-023-03368-y

**Published:** 2023-12-08

**Authors:** Nur Rasyid, Soefiannagoya Soedarman

**Affiliations:** Department of Urology, Faculty of Medicine, Cipto Mangunkusumo Hospital, University, Jakarta Pusat, Indonesia

**Keywords:** Gene polymorphism, Genetic, Urolithiasis, Single nucleotide polymorphisms

## Abstract

**Introduction:**

Urolithiasis is one of the most prevalent diseases worldwide. Its prevalence is rising, both in developing and developed countries. It is known that genetic factors play big roles in the development of urolithiasis. One of the suspected factors is gene polymorphism. This study aims to find an accurate estimate of the association between genetic polymorphism and the risk of recurrent urolithiasis.

**Methods:**

A systematic review and meta-analysis were performed on 12 studies from 3 databases that investigated gene polymorphism as an risk factor of urolithiasis. The review was done using Review Manager® version 5.3.

**Results:**

Insignificant heterogenicity was found in this study. Populations from Asia and the Middle East are more likely to experience recurrent urolithiasis. Additionally, variation in the VDR and urokinase genes, particularly in the Asian population, increases the risk of developing recurrent urolithiasis.

**Conclusions:**

Gene polymorphisms have significant roles in the development of urolithiasis, especially in the Middle Eastern region.

## Introduction

Currently, the incidence and prevalence of urolithiasis is on the rise worldwide [1]. Approximately 12% population of the world was affected by urolithiasis regardless of age, race, or sex [2]. The prevalence of urolithiasis is considerably high in Asia ranging from 5 to 19.1% in various areas including developed countries such as South Korea and Japan [3]. Uniquely, East and North Asia has a lower prevalence of merely 1–8%.^3^ Despite its high prevalence, recent technological advances have allowed urolithiasis to be treated with medication or minimally invasive procedures such as shock wave lithotripsy, percutaneous nephrolithotomy, and ureteroscopy [4]. However, the main issue of urolithiasis is it has a considerably high recurrence rate in 40–50% of affected persons [3, 5]. The recurrence rate can increase to as high as 75% for patients who did not apply for metaphylaxis in 20 years [2]. The high recurrence rate can impact the quality of life and increases the period of follow up which poses a financial burden to those affected. Moreover, there have been cases where a nephrectomy was performed in urolithiasis with a severe urinary infection which, in the worst scenario, may lead to complications such as sepsis [4].

Researchers, through numerous epidemiological studies, have far known that the occurrence of urolithiasis is hereditary related [6]. Numerous candidate genes associated with urolithiasis have been discovered such as genes responsible for the receptor (e.g. vitamin D receptor or calcium-sensing receptor), ion channel (e.g. Claudin 16 or Claudin 19), transporter (e.g. sodium phosphate co-transporter), calcium channel (e.g. transient receptor potential cation channel subfamily V member 5 and member 6), chloride/H^+^ antiporter (e.g. CLCN5), β-glucuronidase (e.g. KLOTHO) and bicarbonate exchanger (e.g. soluble adenylate cyclase) [7]. These genes predominantly lead to increased calcium concentration in the urine and increase the chance of calcium stone formation [7].

Although various genes responsible for urolithiasis have been mapped extensively, the risk of urolithiasis remains unknown let alone how urolithiasis recurrence could occur [8]. Researchers have since funneled down the search into the involvement of genetic polymorphism [9]. Genetic polymorphism is a DNA variant that occurs in a small number of populations. GP arose through mutation and hence the terms polymorphism and mutation can be intertwined. Allele with more than 1% frequency in the general population is defined as polymorphism while an allele frequency less than 1% is called mutation [10]. A type of polymorphism, called single nucleotide polymorphisms (SNPs), is the most frequent type of genetic variation. SNPs are a single base-pair difference variation within the DNA sequence and mostly do not affect an individual’s health. However, some SNPs close to the regulatory region of a gene may affect the gene’s function or an individual’s response to environmental factors. As both recurrent urolithiasis and SNPs are also being inherited from parents, the involvement of genetic polymorphism has shed new light on finding the risk of recurrent urolithiasis [11, 12].

Due to the huge variety of genetic polymorphisms and their small prevalence in the population, the journey to locate these genetic polymorphisms as the risk of recurrent urolithiasis can be challenging. Therefore, this systematic review and meta-analysis are performed to obtain eligible studies to provide a more accurate estimate of the association between genetic polymorphism and the risk of recurrent urolithiasis.

## Patients & methods

### Search strategy

The study was conducted through a comprehensive search from Medline/PubMed, Scopus, and Cochrane electronic databases for studies published between January 2000 and June 2023. We used the search terms: ‘urolithiasis’, ‘nephrolithiasis’, ‘recurrent’, ‘calculi’, ‘stone former’, ‘bladder stone’, ‘kidney stone’, and ‘polymorphism’. The list of references in the included study was manually searched for additional studies.

### Selection criteria

Two investigators (NR & SS) independently performed the study selection. The studies were selected manually for duplication. Duplication-free articles were further examined using predetermined inclusion and exclusion criteria from the titles and abstracts of the articles. The inclusion criteria were considered eligible when the studies (both observational and experimental) include adults (aged more than 18 years) who had at least more than one case of stone formation along the urinary tract condition. Moreover, the odds ratio (OR) estimate and 95% confidence interval (CI) with a significant p-value (p < 0.05) should be reported or at the very least data to calculate the OR is provided.

Studies were excluded if they were case reports or studies conducted on other than humans. Studies that did not discuss recurrent urolithiasis or polymorphisms that did not relate to recurrent urolithiasis will also be excluded. No other restrictions were being imposed. Any differences in opinions were discussed and agreement was reached by consensus.

### Data extraction

The manuscripts, after undergoing intense selection, were then reviewed by two investigators (NR & SS) independently. The information collected from each study includes the first author’s name, the year of publication, the location, the sample size, the age, the polymorphic gene, and the alleles.

### Risk of bias assessment and statistical methods

The risk of bias was assessed by two authors (NR & SS) independently. Bias assessment was done using Strengthening the Reporting of Observational Studies in Epidemiology (STROBE) guidelines for observational studies (Table 1). The bias assessment will not affect studies included in the meta-analysis.

**Table 1:** STROBE analysis of each studies

			Shakhssalim, 2010	Aykan, 2015	Lai KC, 2010	Chou YH, 2010	Tsai FJ, 2002	Chen WC(CTR), 2001	Chen WC (IL-1b), 2001	Yamate, 2000	Eposito, 2017	Huang, 2005	Rendina, 2004	Mossetti, 2003
Title and abstract	1	(a) Indicate the study’s design with a commonly used term in the title or the abstract	V	V	V	V	V	V	X	V	X	X	X	V
		(b) Provide in the abstract an informative and balanced summary of what was done and what was found	V	V	V	V	V	V	V	V	V	V	V	V
**Introduction**														
Background/rationale	2	Explain the scientific background and rationale for the investigation being reported	V	V	V	V	V	V	V	V	V	V	V	V
Objectives	3	State specific objectives, including any prespecified hypotheses	V	V	V	V	V	V	V	V	V	V	V	V
**Methods**														
Study design	4	Present key elements of study design early in the paper	V	V	V	V	V	V	V	V	X	X	X	V
Setting	5	Describe the setting, locations, and relevant dates, including periods of recruitment, exposure, follow-up, and data collection	X	X	X	X	X	V	X	X	X	X	X	X
Participants	6	(a) Give the eligibility criteria, and the sources and methods of case ascertainment and control selection. Give the rationale for the choice of cases and controls	V	V	V	V	X	V	V	V	X	V	V	V
		(b) For matched studies, give matching criteria and the nmber of controls per case	V	V	V	V	X	X	X	X	V	X	V	X
Variables	7	Clearly define all outcomes, exposures, predictors, potential confounders, and effect modifiers. Give diagnostic criteria, if applicable	V	V	V	V	V	V	X	V	V	V	X	V
Data sources/ measurement	8*	For each variable of interest, give sources of data and details of methods of assessment (measurement). Describe comparability of assessment methods if there is more than one group	V	V	V	V	V	V	V	V	V	V	V	X
Bias	9	Describe any efforts to address potential sources of bias	V	V	V	V	X	X	X	X	V	X	V	V
Study size	10	Explain how the study size was arrived at	V	V	V	V	V	V	V	V	V	X	V	V
Quantitative variables	11	Explain how quantitative variables were handled in the analyses. If applicable, describe which groupings were chosen and why	V	V	V	V	V	V	V	V	X	V	X	V
Statistical methods	12	(a) Describe all statistical methods, including those used to control for confounding	V	V	V	V	V	V	V	X	V	X	V	X
		(b) Describe any methods used to examine subgroups and interactions	X	V	X	X	X	X	X	X	X	X	X	V
		(c) Explain how missing data were addressed	X	X	X	X	X	X	X	X	X	X	X	X
		(d) If applicable, explain how matching of cases and controls was addressed	V	V	V	X	X	X	X	X	X	V	X	X
		(e) Describe any sensitivity analyses	X	X	X	X	X	X	X	X	V	X	X	X
**Results**														
Participants	13*	(a) Report numbers of individuals at each stage of study—eg numbers potentially eligible, examined for eligibility, confirmed eligible, included in the study, completing follow-up, and analysed	V	V	V	V	V	V	V	V	X	X	X	V
		(b) Give reasons for non-participation at each stage	X	X	X	X	X	X	X	X	X	X	X	X
		(c) Consider use of a flow diagram	X	X	X	X	X	X	X	X	X	X	X	X
Descriptive data	14*	(a) Give characteristics of study participants (e.g. demographic, clinical, social) and information on exposures and potential confounders	V	V	V	X	X	X	X	X	V	X	X	X
		(b) Indicate number of participants with missing data for each variable of interest	X	X	X	X	X	X	X	X	X	V	V	V
Outcome data	15*	Report numbers in each exposure category, or summary measures of exposure	V	V	V	V	V	V	V	V	V	V	V	X
Main results	16	(a) Give unadjusted estimates and, if applicable, confounder-adjusted estimates and their precision (e.g., 95% confidence interval). Make clear which confounders were adjusted for and why they were included	X	X	X	X	X	X	X	X	V	X	V	V
		(b) Report category boundaries when continuous variables were categorized	V	V	V	V	V	V	V	V	X	X	X	V
		(c) If relevant, consider translating estimates of relative risk into absolute risk for a meaningful time period	X	X	X	X	X	X	X	X	X	X	X	X
Other analyses	17	Report other analyses done—eg analyses of subgroups and interactions, and sensitivity analyses	X	V	X	V	X	X	X	X	V	V	V	X
**Discussion**														
Key results	18	Summarise key results with reference to study objectives	V	V	V	V	V	V	V	V	V	V	V	V
Limitations	19	Discuss limitations of the study, taking into account sources of potential bias or imprecision. Discuss both direction and magnitude of any potential bias	X	V	V	X	X	X	X	X	V	X	X	V
Interpretation	20	Give a cautious overall interpretation of results considering objectives, limitations, multiplicity of analyses, results from similar studies, and other relevant evidence	X	V	V	V	V	V	V	V	V	V	V	X
Generalisability	21	Discuss the generalisability (external validity) of the study results	X	V	V	X	X	V	X	X	V	V	V	V
**Other information**														
Funding	22	Give the source of funding and the role of the funders for the present study and, if applicable, for the original study on which the present article is based	V	X	V	X	X	V	X	V	X	X	X	X

All analysis was performed using Review Manager version 5.3. Meta-analysis was performed when there were at least two similar genetic studies. The strength of association between the polymorphisms in subjects was measured using OR and 95% CI. The OR was calculated for the allelic frequency of the SNP. The statistical significance of OR was measured by using the p-value from the Z test. This model was used to compare the allelic variation within SNP to measure the ratio of recurrence in individuals with certain alleles. The heterogeneity between studies was measured with the Chi-square test Cochrane Q-test and inconsistency index (I^2^) test [13]. The I^2^ value of ≥ 50% was assigned as high heterogeneity, meanwhile, values < 50% were assigned as low heterogeneity. High heterogeneity would be analyzed using random effect, meanwhile, low heterogeneity would be analyzed with fixed effect [14]. P values less than 0.05 were considered to be significant. Publication bias was assessed by using a funnel plot [15].

## Result

### Literature search and characteristics of studies

**Fig. 1:**
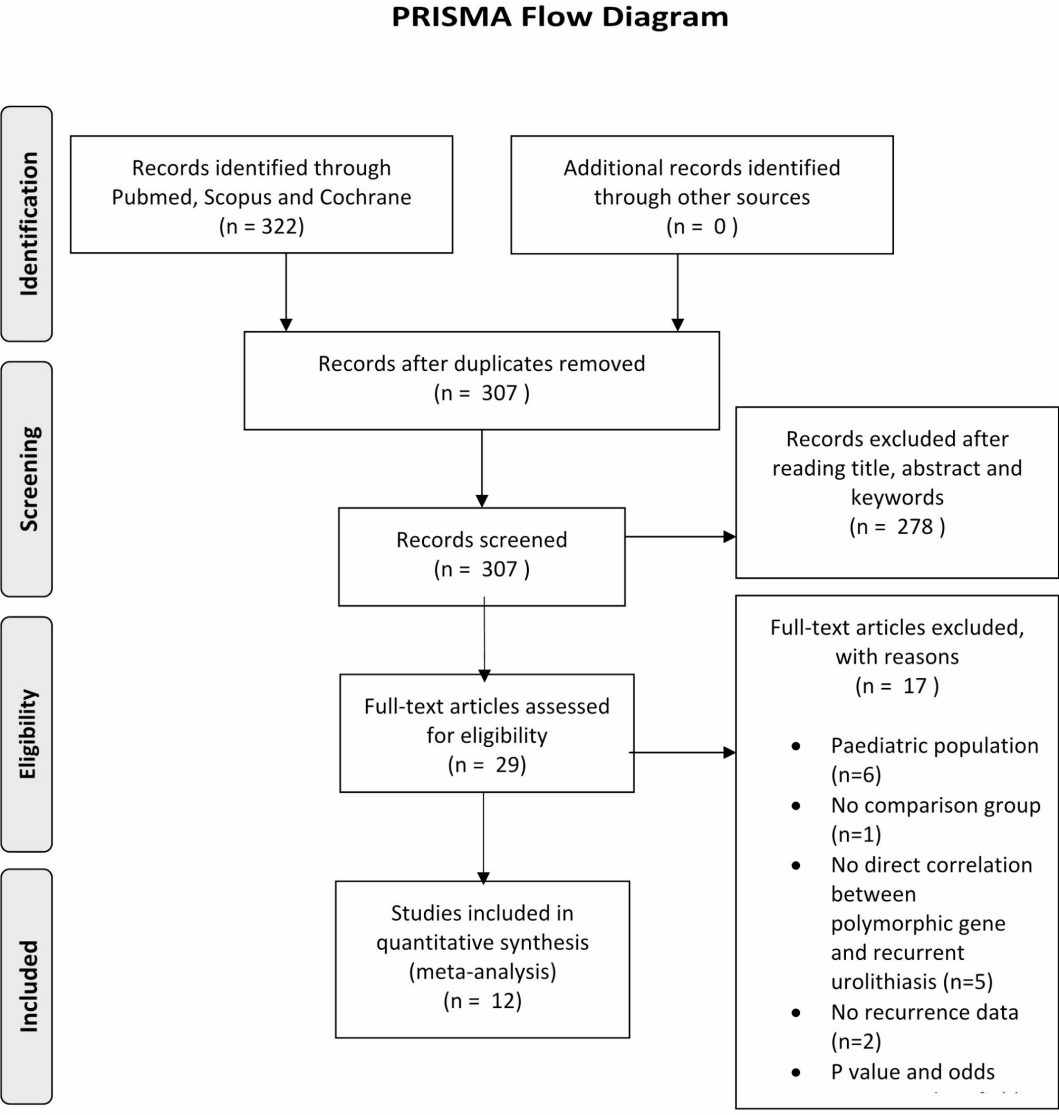
The flow of study included in the meta-analysis

There were a total of 322 articles initially identified by using the search terms and methodology described above. After reviewing the title and abstract of each article by using the inclusion and exclusion criteria, 293 articles were excluded and 29 articles were selected. All these twenty-nine studies were further assessed and 17 articles were considered irrelevant. Finally, there were only 12 case-control studies that met our criteria and were included in the meta-analysis. The details of the study selection process are depicted in Fig. 1.

The twelve selected studies contained 1773 recurrent urolithiasis patients (mean age range ± standard deviation: 40.2 ± 12.0 to 53.87 ± 9.83) and 1946 non-stone former participants (mean age range ± standard deviation: 38.4 ± 6.9 to 53.2 ± 9.9). Three studies were conducted in Italy, two were in the Middle East (Iran and Turkey), and seven were in Eastern Asia (Taiwan and Japan). There were three studies on vitamin D receptor (VDR), two on urokinase, one on calcitonin receptor, one on a calcium-sensing receptor, one on interleukin-18, one on interleukin-1Ra, one on melatonin receptor 1 A, one on ORAI 1, one on osteopontin, and one on TAP2-2. STROBE quality assessment ranging from 10.73 to 17.7. The characteristics of each study are presented in Table 2.

**Table 2:** Characteristics of the case-control studies included in the meta-analysis

Author	Year	City/ Country	Region	Gene (SNP)	Grouping (M/F/ Total)	Mean Age ± Standard Deviation (years)	Inclusion Criteria	Exclusion Criteria
Mossetti G	2003	Naples,Italy	Europe	Vitamin D Receptor	Cases (128/92/ 220)	41.09 ± 14	Recurrent stone forming patients with two or more calcium stones in the past 4 years	Urinary tract infections (UTI), hyperparathyroidism, cystinuria, gouty diathesis, renal tubular acidosis, low creatinine clearance, chronic diarrhoeal states, intake of thiazide diuretics, angiotensin-converting enzyme (ACE)inhibitors, glucocorticoids or oestrogens
					Controls (63/51/ 114)	40.37 ± 14.07	Unrelated healthy subjects without history of nephrolithiasis	presence of one or more metabolic risk factor for nephrolithiasis
Rendina D	2004	Naples,Italy		Vitamin D Receptor	Cases (94/65/ 159)	43.2 ± 10.9	Unrelated patients with recurrent stone formation with 2 or more calcium stones within previous 4 years and idiopathic hypercalciuria.	Gouty diathesis; cystinuria; renal tubular acidosis; low creatinine clearance; debilitating physical illnesses; hyperthyroidism; primary hyperparathyroidism, Paget’s bone disease, urinary infections, use of corticosteroid, diuretics, NSAID, vitamin D, or lithium.
					Controls (72/52 /124)	41.9 ± 10.4	Unrelated healthy subjects without history of nephrolithiasis.	presence of idiopathic hypercalciuria with a nonrestricted diet.
Esposito T	2017	Naples, Italy		Melatonin Receptor 1 A	Cases (136/110/236)	40.2 ± 12.0	Idiopathic recurrent calcium stone former with at least 2 or more history of calcium oxalate stone	Exclusion criteria is the same with (Mosseti G, 2003)
					Controls (141/128/269)	40.3 ± 11.8	Healthy without history of nephrolithiasis	
Shakhssalim N	2010	Tehran/Iran	Middle East	Calcium-Sensing Receptor	Cases (99/-/99)	43.4 ± 6.9	Idiopathic recurrent calcium kidney stone-forming men with 2 symptomatic episodes at least 6 months apart during the past 5 years	history of metabolic, gastrointestinal, hepatic, renal, or endocrinological disease
					Control (99/-/107)	38.4 ± 6.9	Healthy volunteer men in the same age range	
Aykan S	2015	Istanbul/ Turkey		Urokinase & Vitamin D Receptor	Cases (50/28/ 78)	41	Recurrent urolithiasis	Patients taking vitamin D and/or calcium supplement.
					Controls (87/80/ 167)	45	Healthy subjects with normal urinalysis and absence of stone in ultrasound study	Another exclusion criteria for control group were patients with family history of urolithiasis.
Yamate T	2000	Osaka/ Japan		Osteopontin	Cases (32/8/ 40)	50.4	Recurrent calcium-containg calculi at least 2 or more episodes	
					Controls (20/16/ 36)	54.3	Normal subjects without past history of urolithiasis	
Chen WC	2001	Taichung/ Taiwan	Eastern Asia	Calcitonin Receptor	Cases (72/30/ 102)	44.6 ± 12.05	Recurrent calcium oxalate stone	hypercalcemia, hyperuricemia, and hyperuricosuria, and urinary tract infections.
					Controls (60/45/ 105)	53 ± 10.08	Healthy volunteers with no history of stone disease or renal calcification	Urinary microscopic hematuria
Chen WC	2001	Taichung/ Taiwan		Interleukin-1Ra	Cases (117/35/152)	44.62 ± 12.05	Recurrent calcium oxalate stone	Urinary tract infection during period of stone treatment
					Controls (-/-/105)	> 40	Healthy volunteers who had no history of familial stone disease or renal calcification	Urinary microscopic hematuria
Tsai FJ	2002	Taichung/ Taiwan		Urokinase	Cases (118/35/153)	44.2 ± 12.0	Recurrent calcium oxalate stone of at least 2 episodes regardless of family history of stone disease	Symptoms of urinary tract infection
					Controls (65/40/ 105)	54.7	Healthy volunteers who had no history of familial stone disease or cancer	Urinary microscopic hematuria
Huang SH	2005	Taichung, Taiwan		TAP2-2	Cases (158/50/208)	43.8 ± 11.7	Recurrent idiopathic calcium oxalate stone disease regardless of family history	Patients with hypercalcemia, hyperuricemia, hyperuricosuria, and symptoms of urinary tract infections
					Controls (147/63/210)	53.2 ± 9.9	Healthy volunteer over the age of 40 who had no familial history of stone disease	Patients with microscopic hematuria
Lai KC	2009	Taichung/ Taiwan		Interleukin-18	Cases (182/90/272)	43.8 ± 11.7	Recurrent idiopathic calcium stone oxalate stone disease regardless of family history	Hypercalcemia, hyperuricaemia, or hyperurocosuria, and urinary tract infection
					Controls (73/31/ 104)	53.2 ± 9.9	Age- and gender- matched healthy volunteers with no familial history of stone disease	Patients with microscopic hematuria
Chou YH	2011	Kaohsiung/ Taiwan		ORAI 1	Cases (34/20/ 54)	53.87 ± 9.83	At least two symptomatic episodes at least 6 months apart or new stones after treatment	Patients with noncalcium renal stone
					Controls (289/211/500)	49.5 ± 15.5	Normal urinalysis, no history of familial stone disease, and no renal calcification history	

Some of the studies discussed multiple SNPs within the polymorphic gene. We identified these SNPs along with their particular alleles. Each genotype consists of 2 alleles which we categorized into “allele 1” and “allele 2”. The categorization was used because “allele 2” was deemed to be the allele that caused recurrent urolithiasis. It is evidenced that the OR of most allele 2 showed significantly higher prevalence within the recurrent urolithiasis population compared to 1(p < 0.05). The summary of the studies is shown in Table 3.

**Table 3:** The list of the polymorphic gene, the specific single nucleotide polymorphism (SNPs), its particular allele and the odds ratio (OR) that suspected as risk of recurrent urolithiasis

Author	Polymorphic gene	SNP	Allele 1 (case/control)	Allele 2 (case/control)	OR of Allele 2	CI	p-value
Chen WC	Calcitonin receptor	*AluI*	C (175/204)	T (29/6)	5.634 [2.286, 13.885]	95%	< 0.01
Shakhssalim (a)	Calcium Sensing Receptor	*R990G*	R (184/212)	G (14/2)	8.06 [1.80, 35.9]	95%	0.006
Shakhssalim (b)	Calcium Sensing Receptor	*A986S*	A (168/200)	S (30/14)	2.55 [1.31, 4.96]	95%	0.006
Lai KC	Interleukin-18	*TaqI*	A (266/185)	C (137/23)	4.14 [2.56, 6.69]	95%	< 0.001
Chen WC	Interleukin-1Ra	*VNTR*	Type II (3/12)	Type I (293/194)	9.041 [1.683, 21.687]	95%	0.005
Esposito T (a)	Melatonin Receptor 1 A	*rs13140012*	A (342/317)	T (150/221)	0.63 [0.49, 0.81]	95%	0.0004
Esposito T (b)	Melatonin Receptor 1 A	*rs6553010*	C (313/383)	T (179/155)	1.41 [1.09, 1.84]	95%	0.009
Chou YH	ORAI 1	*rs12313273*	T (62/750)	C (46/250)	2.23 [1.48, 3.35]	95%	0.037
Yamate T	Osteopontin	*AluI*	C (28/38)	T (52/34)	2.08 [1.08, 3.98]	95%	< 0.05
Huang SH	TAP2-2	*MspI*	G (169/246)	A (245/170)	2.10 [1.59, 2.77]	95%	< 0.0001
Aykan S (b)	Urokinase	*ApaLI*	C (133/310)	T (23/24)	2.23 [1.22, 4.10]	95%	0.01
Tsai FJ	Urokinase	*ApaLI*	C (288/206)	T (18/4)	3.088 [1.090, 8.995]	95%	0.028
Aykan S (a)	Vitamin D receptor	*TaqI*	T (80/218)	C (76/116)	1.79 [1.21, 2.63]	95%	0.003
Rendina D (a)	Vitamin D Receptor	*ApaI*	A (173/142)	a (145/106)	1.12 [0.80, 1.57]	95%	< 0.05
Rendina D (b)	Vitamin D Receptor	*BsmI*	B (163/134)	b (155/114)	1.12 [0.80, 1.56]	95%	< 0.05
Mossetti G (a)	Vitamin D receptor	*TaqI*	T (264/136)	t (176/92)	0.99 [0.71, 1.37]	95%	< 0.001
Mossetti G (b)	Vitamin D receptor	*BsmI*	B (202/122)	b (238/106)	1.36 [0.98, 1.87]	95%	0.005

### Quantitative data synthesis

**Fig. 2:**
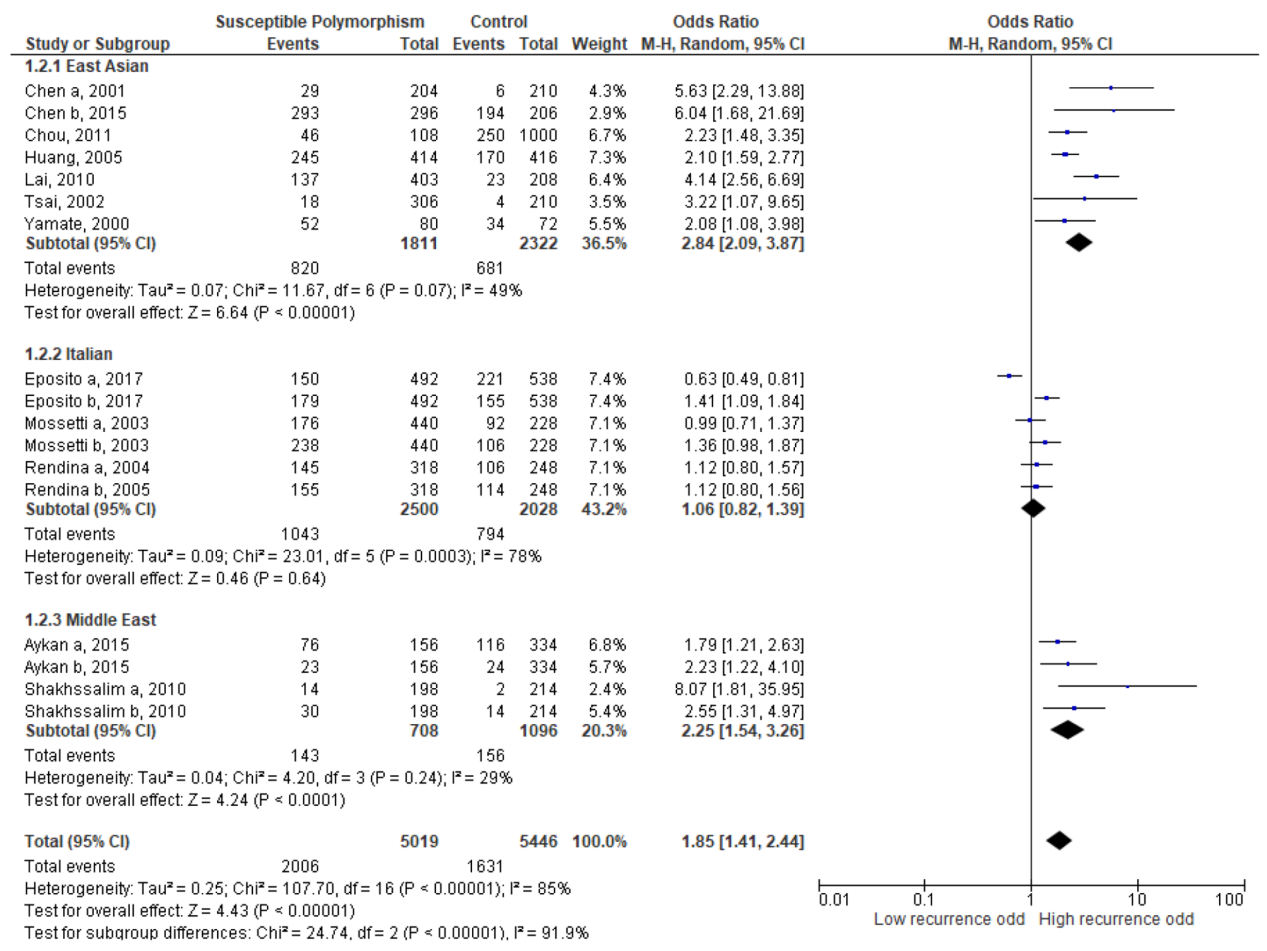
Meta-analysis for the association between genetic SNP and recurrent urolithiasis

As shown in Fig. 2, our meta-analysis of genetic factors of recurrent urolithiasis showed significant heterogeneity (I^2^ = 85%, p < 0.00001). Therefore we used random effect for the analysis of all genetic studies. Meta-analysis of genetic effect on recurrent urolithiasis involved 12 studies, among 5019 allele frequency in recurrent stone formers and 5446 allele frequency in controls. We found 14 SNPs within 10 genes had a significant association with recurrent urolithiasis (Fig. 2, overall OR = 1.85, 95% CI = 1.41–2.44, p < 0.0001). Subgroup analysis was done to reduce heterogeneity. In the East Asian population, we found a significant association between genetic polymorphism and recurrent urolithiasis (OR = 2.84, 95% CI = 2.09–3.87, p < 0.00001) and there was no significant decrease in heterogeneity value (I^2^ = 49%, p = 0.07). In the Italian population, there was no significant association between genetic polymorphism and recurrent urolithiasis (OR = 1.06, 95% CI = 0.82–1.39, p = 0.64) with high heterogeneity (I^2^ = 78%, p = 0.0003). Meanwhile, in the Middle East population, there was a significant association between genetic polymorphism and recurrent urolithiasis (OR = 2.25, 95% CI = 1.54–3.26, p < 0.0001) with no significant decrease of heterogeneity (I^2^ = 29%, p = 0.24). We found a significant difference between each population subgroup (I^2^ = 91.9%, p < 0.00001).

### Vitamin D receptor and urokinase gene polymorphism

**Fig. 3:**
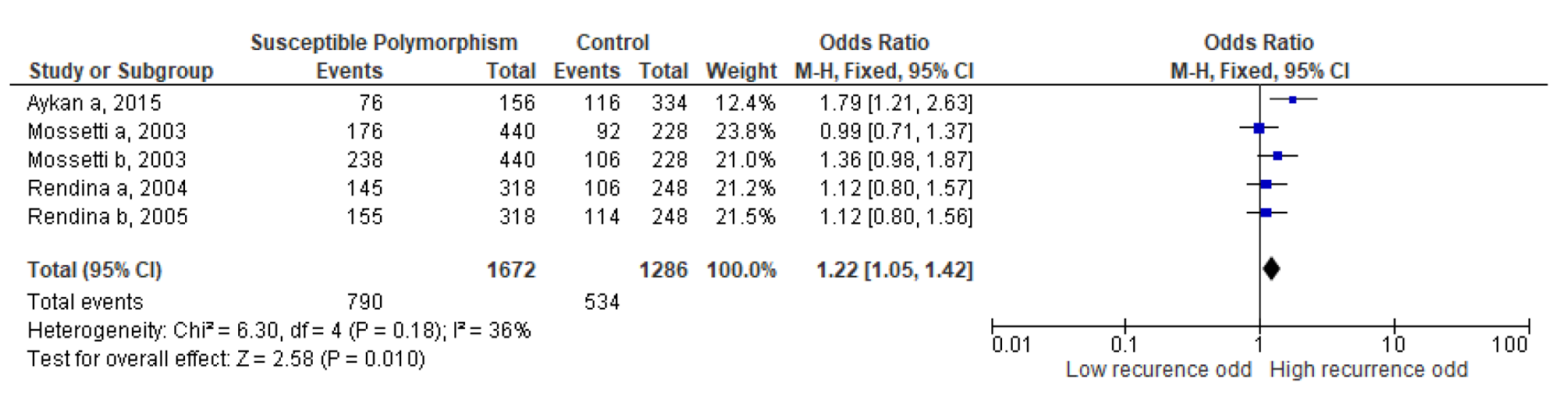
Meta-analysis of vitamin D receptor gene polymorphism and recurrent urolithiasis

The meta-analysis of the VDR gene involving 5 studies with a total of 1672 allele frequency in recurrent stone formers and 1286 allele frequency in controls, also showed a significant association with recurrent urolithiasis (Fig. 3, overall OR = 1.22, CI 95% = 1.05–1.42, p = 0.03). We found no publication bias within the studies included in the analysis of VDR gene SNP.

**Fig. 4:**
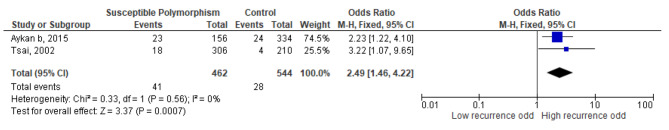
Meta-analysis of urokinase gene polymorphism and recurrent urolithiasis

Two studies were included for the polymorphism of the urokinase gene. There were a total of 462 recurrent stone formers and 544 controls. We found a strong association between polymorphism of the urokinase gene and recurrent urolithiasis (Fig. 4. OR = 2.49, 95% CI = 1.46–4.22, p = 0.0007) with no significant heterogeneity (I^2^ = 0%, p = 0.56). There’s no publication bias in the included studies.

## Discussion

In this meta-analysis, we aim to find the correlation between various genetic polymorphisms with recurrent urolithiasis. To the best of our knowledge, this is the first meta-analysis to investigate the association between recurrent urolithiasis risk and multiple gene polymorphisms. Overall, individual bearing the allele correlated with recurrent urolithiasis has an increased risk by 1.85 times to develop recurrent urolithiasis. There was also high heterogeneity within the included studies (I^2^ = 85%). Therefore, subgroup analysis was performed based on the populations (e.g. East Asian, Italian, and Middle Eastern). We found that genetic polymorphism in the East Asian population showed the highest risk of developing recurrent urolithiasis with an increased risk of 2.84 times with no significant heterogeneity (I^2^ = 49%), followed by the Middle East population with an increased risk of 2.25 times also with no significant heterogeneity (I^2^ = 29%). However, the risk of developing recurrent urolithiasis in the Italian population is little or none with high heterogeneity (I^2^ = 79%). These findings were probably caused by the difference in genetic polymorphism within the population. The difference between the Asian population (East Asia and the Middle East) with the Italian might be due to the number of allele frequencies included, especially in the Middle East population (708 cases and 1096 controls). There might be some other causes of differences within ethnicity such as other factors affecting the genetic polymorphism. Therefore, an epidemiological study might be needed to investigate probable factors.

When considering the potential impact of various SNPs, we performed analyses on VDR and urokinase gene polymorphisms. Our result showed that VDR gene polymorphism increases the risk of recurrent urolithiasis by 1.22 times with no significant heterogeneity (I^2^ = 36%). The SNPs for VDR genes included in this study were *BsmI, ApaI, and TaqI*. All of these SNPs are located at the 3’ UTR region of VDR gene mRNA and correlate with increased stability and higher vitamin D activity [16, 17]. A number of meta-analyses have documented that *ApaI and TaqI* polymorphisms are known to be associated with urolithiasis [18, 19]. While, some other studies highlighted *TaqI, ApaI* and *BsmI* SNPs in the Asia population are more prone to develop urolithiasis [18, 20]. Accordingly, a larger and more comprehensive study was performed. The study concluded that *TaqI* polymorphism in Asians increased the risk of urolithiasis, but not in Caucasians [19]. Although all this research had shown *TaqI* polymorphism to be closely related to Asians, a recent meta-analysis opposed the previous claims and reported no association between *TaqI, ApaI*, and *BsmI* with urolithiasis in the Asian population [21]. Another recent meta-analysis claimed with mixed results regarding these SNPs association with Asians and Caucasians population [22]. Unfortunately, our study is unable to conclude the involvement between VDR gene polymorphism and urolithiasis recurrence in either the Asian or Caucasian population as the population obtained for our VDR gene polymorphism analysis study is largely derived from European countries. To date, there are still inconclusive results regarding the involvement of VDR SNPs in urolithiasis let alone its recurrence.

In the urokinase gene subanalysis, only a single SNP, *ApaLI* with T allele, was successfully collected. Individuals possessing the urokinase gene “T” allele showed a higher risk of developing urolithiasis recurrence (OR = 2.49) with no significant heterogeneity (I^2^ = 0%) than VDR gene polymorphism. Urokinase prevents the breakdown of the protein matrix within the stone and retains the formation of the stone [23]. Urokinase gene is located at chromosome 10q22.2 where a few SNPs are also located in this gene [23]. Of these multiple sites, 3’-UTR T/C polymorphism at the + 4065 nucleotide is the most commonly studied polymorphic site [24]. *ApaLI* is one of the SNP located at this site [25]. Nevertheless, study regarding *ApaLI* is still limited and all studies available are located in Asia. Preceding our study, a meta-analysis confirmed that urokinase 3’UTR T/C polymorphism is linked with urolithiasis in the Asian population, thereby confirming our findings that urokinase gene polymorphism can cause both single and recurring urolithiasis [24].

Urolithiasis is a multifactorial disease and gene polymorphism is one of the factors [3]. The other suspected risk factor for recurrent urolithiasis is the high level of vitamin D [3]. Vitamin D, obtained from sun exposure, is a hormone that regulates calcium and phosphorus metabolism [26]. Increased vitamin D metabolites and their active form is highly correlated with hypercalciuria urolithiasis and urinary stones, respectively [26]. Moreover, recurrence patient has higher calcium excretion in the urine [27]. Despite the Middle East being bathed with enormous sunlight all year long, due to clothing behavior, up to 80% of Middle East countries are vitamin D deficient [28]. Similarly, vitamin D deficiency can also be found in East Asia countries [29, 30]. These findings curb our suspicion in the relation of vitamin D level with recurrent urolithiasis. Nevertheless, more correlation studies were needed to confirm this claim. Another risk factor is a hot dry climate and low liquid intake that lead to low urine volume (< 2 L/day) [31]. Tropical and sub-tropical countries have a higher prevalence of urolithiasis notably in the summer [3]. For example, the Arab countries with their humid and hot climate exaggerated with people with limited fluid intake has led to a small volume and highly concentrated 24-hour urine [32].

We should consider the effect of heterogeneity and publication bias within our analysis. Our overall genetic polymorphism studies showed significant heterogeneity, which we suspect was caused by the difference in population or genetic polymorphism. After the subgroup analysis of the population, we found no significant heterogeneity in the East Asian population and the Middle East population, and also after performing analysis based on the genetic polymorphism (VDR and urokinase) the heterogeneity effectively disappeared. We can conclude that most of the heterogeneity was caused by population and genetic polymorphism differences. We found publication bias in the overall studies with could also impact our result of finding, but analysis based on vitamin D receptor and urokinase gene didn’t show any publication bias.

There were some limitations within our study. The number of studies included in this meta-analysis was considerably small. A lot of relevant published/unpublished studies might be missed out. The studies we included only consisted of East Asian, Italian, and Middle East populations which might not represent all general populations. We also couldn’t analyze the gene-gene interaction and gene-environment interaction due to a lack of information within the included studies. Other risk factors like urinary tract infection, hypercalciuria, or microscopic hematuria also couldn’t be analyzed because most of the studies didn’t provide any information regarding the case and control with associated risk factors. Finally, we only managed to perform an analysis of VDR and urokinase gene polymorphism on recurrent urolithiasis due to a lack of studies regarding other genetic polymorphism and recurrent urolithiasis.

## Conclusion

In conclusion, Asia and Middle Eastern populations have a higher risk of developing recurrent urolithiasis. Additionally, both VDR and urokinase gene polymorphism contributes to the susceptibility of recurrent urolithiasis particularly for the Asian population in the latter. Studies with a variety of population characteristics are recommended to be performed to further support our results.

## Data Availability

The datasets used and/or analyzed during the current study are available from the corresponding author upon reasonable request.
